# Supplementary material for: RBD Double Mutations of SARS-CoV-2 Strains Increase Transmissibility through Enhanced Interaction between RBD and ACE2 Receptor
Source: Viruses. 2021 Dec 21;14(1):1. doi: 10.3390/v14010001 (PMC8781274; doi:10.3390/v14010001)
Supplement: Supplementary file 1 [file viruses-14-00001-s001.zip › viruses-1481745 -suppl figures.pdf]

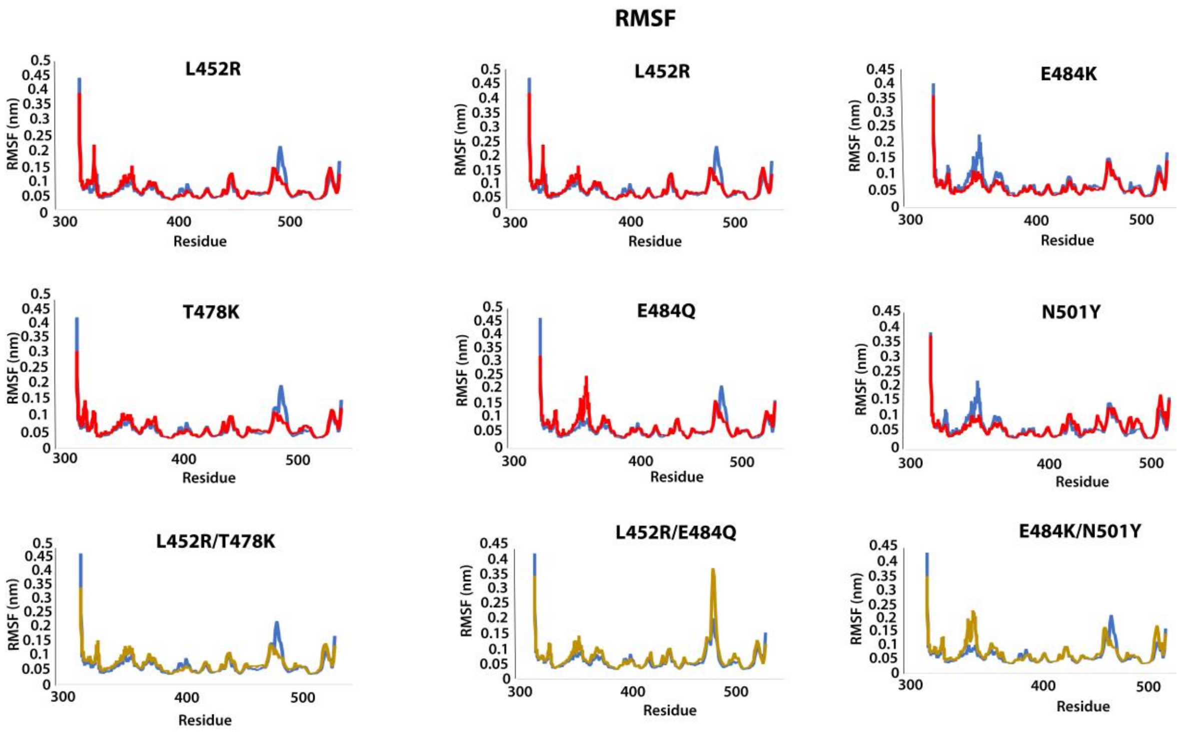

Figure S1. Dynamic changes of RBD structure by RMSF analysis in wild-type, single and double mutant structures. The x-axis represents the residue positions of the RBD domain and y-axis represents the RMSF value for the structures. Blue: wild-type; red: single mutant; yellow: double mutants.

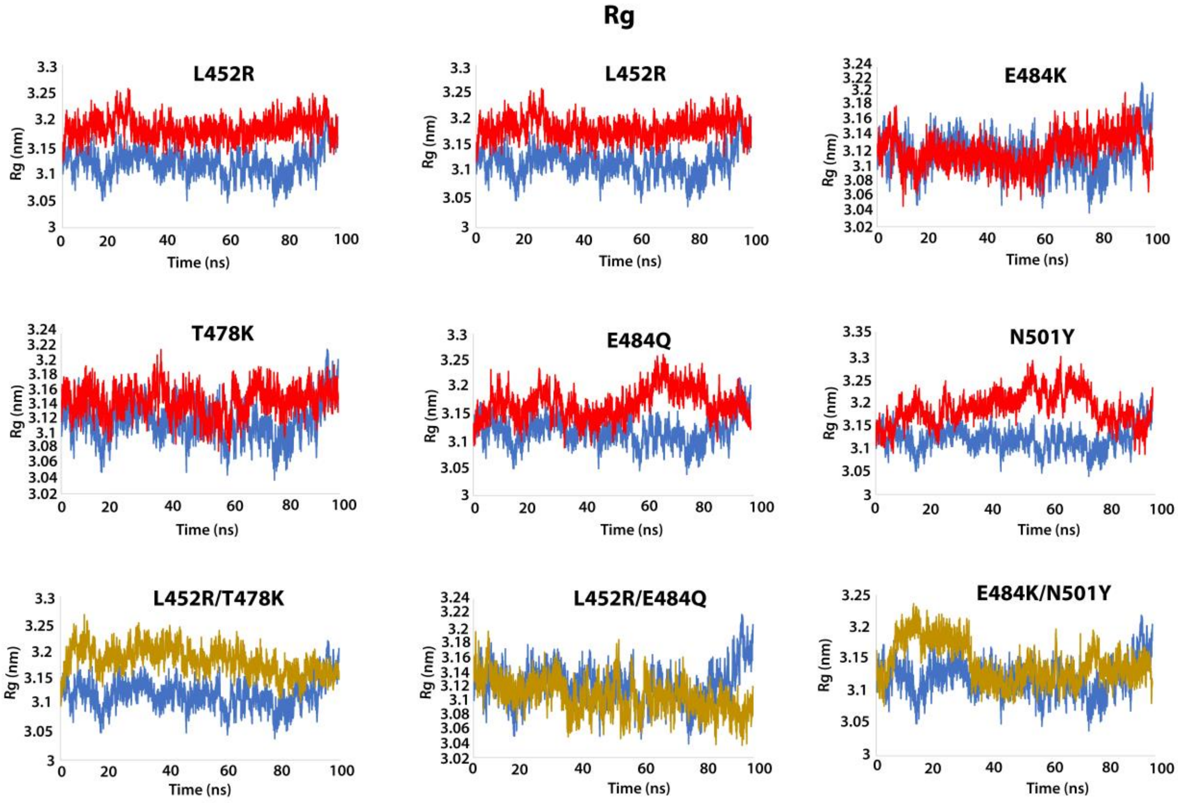

Figure S2. Dynamic changes of RBD structure by Rg analysis in in wild-type, single and double mutant structures. The x-axis represents the time period of 100 ns and y-axis represents the Rg value for the structures. Blue: wild-type; red: single mutant; yellow: double mutants.

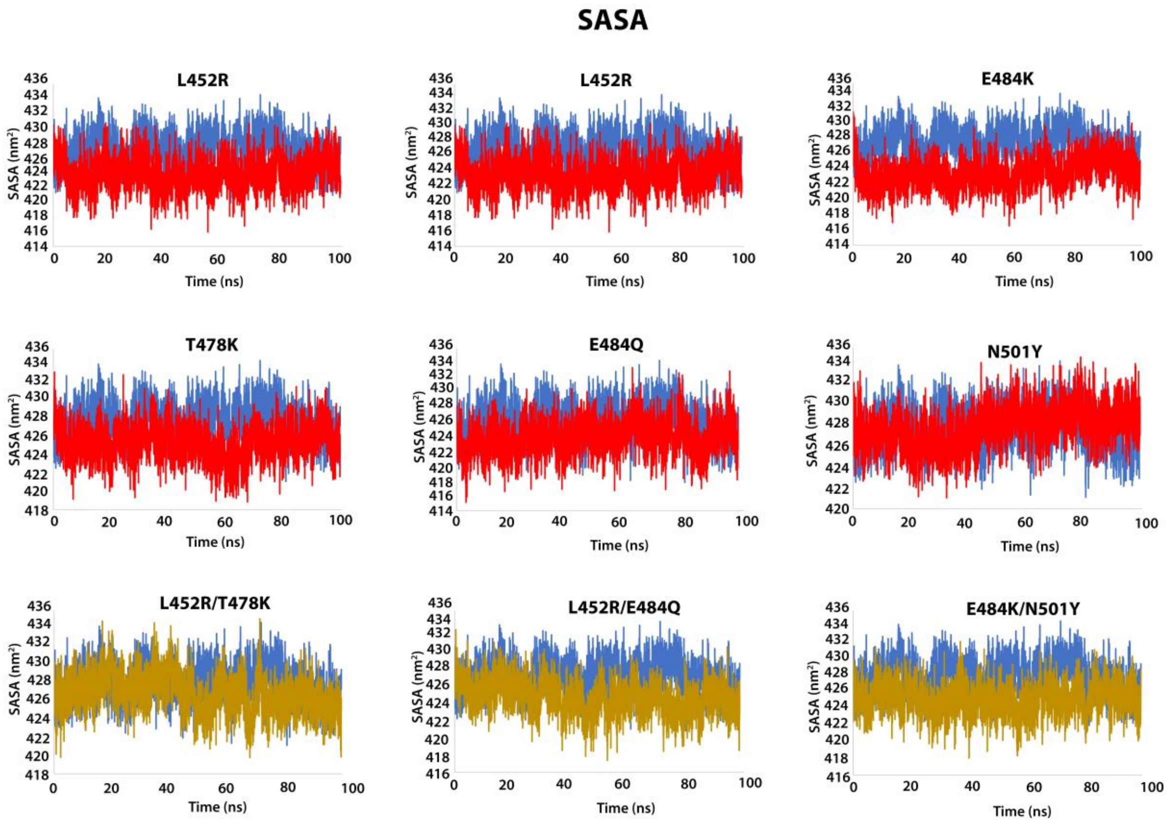

Figure S3. Dynamic changes of RBD structure by SASA analysis in wild-type, single and double mutant structures. The *x*-axis represents the time period of 100 ns and *y*-axis represents the SASA value for the structures. Blue: wild-type; red: single mutant; yellow: double mutants.

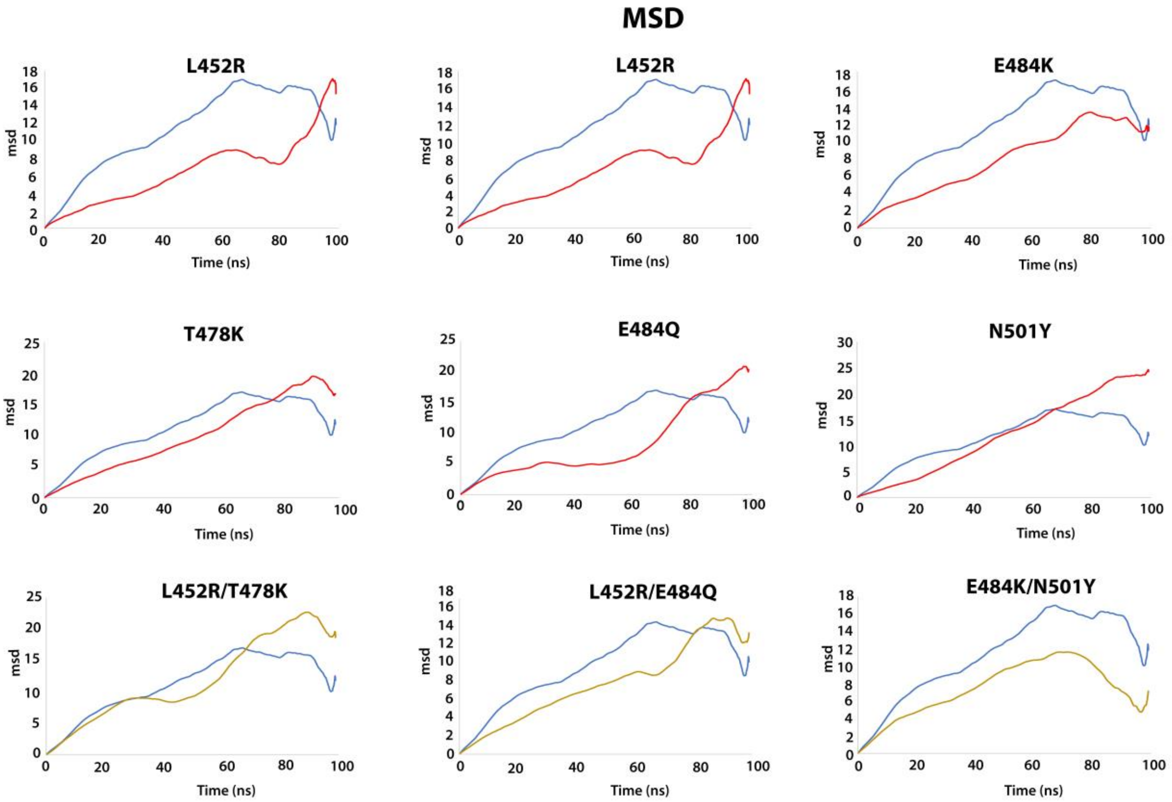

Figure S4. Dynamic changes of RBD structure by MSD analysis in wild-type, single and double mutant structures. The *x*-axis represents the time period of 100 ns and *y*-axis represents the MSD value for the structures. Blue: wild-type; red: single mutant; yellow: double mutants. .
